# Lessons learnt in the response to COVID-19 in Mozambique: enabling readiness for the next pandemic

**DOI:** 10.3389/frhs.2025.1612577

**Published:** 2025-07-31

**Authors:** Mariana E. Posse, Grace Njeri Muriithi, Daniel Malik Achala, Elizabeth Naa Adukwei Adote, Chinyere Ojiugo Mbachu, Senait Alemayehu Beshah, Chijioke Osinachi Nwosu, John Ele-Ojo Ataguba

**Affiliations:** ^1^ Department of Research, Mariana Posse Consultoria, EI, Maputo, Mozambique; ^2^ African Health Economics and Policy Association (AfHEA), Accra, Ghana; ^3^ Department of Community Medicine, University of Nigeria, Enugu Campus, Enugu, Nigeria; ^4^ Department of Economics and Finance, Ethiopian Public Health Institute, Addis Ababa, Ethiopia; ^5^ Department of Economics and Finance, University of the Free State, Bloemfontein, South Africa; ^6^ Department of Community Health Sciences, Max Rady College of Medicine, Rady, Winnipeg, MB, Canada; ^7^ Health Economics Unit, School of Public Health, Faculty of Health Sciences, University of Cape Town, Cape Town, South Africa; ^8^ Partnership for Economic Policy (PEP), Nairobi, Kenya; ^9^ School of Health Systems and Public Health, University of Pretoria, Pretoria, South Africa

**Keywords:** COVID-19, pandemic, Mozambique, response, lessons learned, vulnerable populations, equity, disaster readiness

## Abstract

**Introduction:**

The coronavirus disease 2019 (COVID-19) has led to a dramatic loss of human lives worldwide and caused economic and social disruptions. The risk of another pandemic occurring is ever-present requiring countries to document factors that influenced the response to COVID-19 to guide the response to future pandemics. This study documents lessons learnt from Mozambique's COVID-19 response, considering the perspectives of various stakeholders and examining different components of the response.

**Methods:**

We used a qualitative phenomenology research design and collected data using in-depth interviews. We used purposive sampling by selecting institutions with relevant experience and knowledge to inform the study objectives. We also used snowballing techniques by asking respondents for other potential informants. We interviewed 19 individuals indicated by the representatives of the institutions selected for the study. The institutions were mostly based in Maputo city, the country's capital. Participants were asked about their role in the organization; responsibility in vaccine distribution and delivery in Mozambique; their opinion on what worked well in the country's response to COVID-19, and what could be improved as preparation to future pandemics. Data was coded using a computer-assisted qualitative data analysis software Maxqda 2020 and analyzed using a deductive thematic approach. A validation meeting was held, in which research participants were asked to check the accuracy of the results and interpretations.

**Results:**

Key drivers of the COVID-19 response were strong leadership; a clear plan and strategies; a functional coordination mechanism; the use of evidence to make decisions; a careful consideration of priority groups; investments in the supply chain and surveillance systems; the utilization of pre-existing vaccination structures; and partnership between the government and several stakeholders. There is room for improvement including the development of a clear budget, a communication plan, creation of an emergency fund, accountability in the use of funds, decentralization of surveillance infrastructure and representation of vulnerable, marginalized, and hard-to-reach populations in the design and implementation of pandemic response.

**Conclusion:**

The lessons learned from the COVID-19 response in Mozambique, which could be considered when preparing for an effective and equitable response to future pandemics, are in essence the following: there should be government leadership, a response plan, adequate resources, use of data to inform decisions, constant vigilance, a prompt response, involvement of all stakeholders and documentation of actions for continuous learning. These lessons could improve pandemic preparedness nationally and globally.

## 1 Introduction

The coronavirus disease 2019 (COVID-19) has led to a dramatic loss of human life worldwide and caused economic and social disruption (1). Initially, countries responded with nonpharmaceutical interventions, i.e., social distancing, quarantine, and personal protective equipment, which alone could not end transmission of COVID-19 and were associated with exacerbated existing vulnerabilities (2). The availability of vaccines enabled the control of the COVID-19 by breaking transmission, reducing related illnesses, hospitalizations, and deaths. Therefore, vaccines became the main component of the COVID-19 response.

However, in resource-limited countries, vaccination rates have been low if compared to affluent nations due in part to the limited supply of vaccines. Factors limiting supply include failure to include these countries in decision making, vaccine nationalism, misinformation, and these countries restricted vaccine manufacturing capacity (3). Nonetheless, initiatives such as COVAX (COVID-19 Vaccines Global Access) and direct country donations, helped resource-limited countries obtain the necessary doses (1). Nevertheless, competing priorities from other endemic diseases, weak public health infrastructure, limited human resources, inadequate storage system due to funding issues often lead to expired and inactive vaccines, slowing vaccination efforts. These problems are mostly evident in rural communities and villages due to epileptic electricity and frequent power outage, limited cold chain equipment and facilities, bad road network which hinders movement of vaccines and vaccinators to intending communities (4–6).

Despite those challenges, these countries managed to turn vaccines into vaccinations and control the pandemic. For example, Mozambique managed to vaccinate 97% of the target population (i.e., health professionals, the elderly living in nursing homes and nursing home workers, people with chronic conditions, the defense forces and prisoners) by December 2023 [Ministry of Health Integrated epidemiological surveillance system (MISAU—SIVE)]. The private sector and civil society organizations (CSOs) complemented government efforts to rapidly strengthen and scale COVID-19 response, for example, by supporting demand creation, providing the infrastructure necessary to store, transport COVID-19 vaccines and by supporting data processing to inform policy makers and health managers (7, 8).

Unfortunately, COVID-19 is not the world's last pandemic. For this reason, it is advisable for countries to document what they have learnt in responding to COVID-19 to guide the response to similar future pandemics. Existing COVID-19 evidence in Mozambique focuses on factors associated with acceptance of and hesitancy towards COVID-19 vaccines, reasons for COVID-19 vaccine acceptance and hesitancy, approaches used to reduce hesitancy towards COVID-19 vaccines, costs to deliver vaccines, and on how social protection policies were equitable during the response (9–17). This evidence is important. For example, the evidence on COVID-19 vaccine acceptance and hesitancy can inform strategies to improve trust in vaccines and in health systems and therefore, increase vaccine uptake and coverage. However, a high vaccination coverage and uptake is not only dependent on trust of vaccines and health systems, but also on other components that help ensure that vaccines are available for delivery, such as the planning and coordination of vaccine distribution process. There are studies that focus on some of these components, i.e., the coordination of the national response to COVID-19, in the first year of the pandemic in Mozambique (between March 2020 and February 2021) (18, 19). However, documenting lessons learned on the different components of the response to COVID-19 can help to comprehensively identify what worked well in the response and what could be improved in terms of both effectiveness and equity, contributing therefore with evidence for better preparedness for future pandemics nationally and globally. This study documents lessons learnt from Mozambique's COVID-19 response, considering the perspectives of various stakeholders and examining different components of the response.

Mozambique is a republic and a multiparty democracy. It has an executive president as head of state and government, who is directly elected for a five-year term and serves a maximum of two terms. The president appoints the prime minister and Council of Ministers. The country has an area of approximately 800,000 km2 and a population of 32.4 million people (concentrated in major cities) as of 2024 (20). The country is characterized by sharp contrasts between the north and the south, defined by the geographic division posed by the Zambezi River. In the north, the topography is made up of hills, low plateaus and rugged highlands, while the south is mainly made up of lowlands. In demographic terms, the north has a spatially dispersed population, while the south has population clusters around large urban areas and transport networks (21). According to the 2017 national Census, 51.6% people above five years old were literate (22)

The country's transport infrastructure develops mainly in a west-east direction, connecting mining and agricultural areas to ports. Energy and information and communication technology infrastructure (ICT) networks follow the population, concentrating on the nodes of the transport corridors. Thus, the highest density of electricity and ICT is in the center-south and south zones. In general, infrastructure in Mozambique, especially in rural areas, remains underdeveloped, despite some improvements in essential sectors such as transport and the electricity grid. Access to basic services, including water, sanitation and electricity, is below regional averages. Although the main road network remains functional, secondary roads are often in poor condition, particularly during the rainy season. Economically, the northern region focuses mainly on agriculture and is responsible for the production of most export crops, while the southern region is characterized by industrial and mining activities (21). The average gross domestic product (GDP) per capita was USD 623 in 2023. Mozambique|Data.

The National Health System is structured in the following subsystems: (i) a public subsystem run by the state and called the National Health Service; (ii) a private subsystem; (iii) a military and paramilitary health subsystem; (iv) a socio-professional organizations such as the Medical Council, the Nursing Council, and the Medical Association of Mozambique, considered part of the National Health System. The National Health Service is directly dependent on the Ministry of Health and is by far the largest provider of healthcare to most of the population. It includes 15 central and provincial hospitals, 55 rural and general hospitals, and 1,659 health centers and 112 health posts spread throughout the districts and communities in the country (20). It employs over 90 per cent of health workers.

The private subsystem is divided into (a) private for-profit; and (b) private not-for-profit medicine. The private for-profit sector is concentrated in the cities (mainly in the capital, Maputo) and includes two hospitals (both in Maputo City), a few dozen clinics, medical practices, pharmacies, laboratories, and imaging services. The for-profit private sector is devoted almost exclusively to curative activities. The private not-for-profit subsystem includes religious organizations and non-governmental organizations (NGOs), mostly foreign and directly funded by donors. It also includes health facilities in some large public and private companies and in educational establishments, such as the Eduardo Mondlane University. The military and paramilitary health subsystem is in an early stage. The National Health Institute (aimed at research) and the Traditional Medicine Institute are under the auspices of the Ministry of Health (23).

## 2 Materials and methods

### 2.1 Study design and participants

We used a qualitative phenomenology research design and collected data using in-depth interviews. This approach was chosen because it was the most appropriate to achieve the objectives of the study, i.e., to understand how the response to COVID-19 was implemented, which was context specific and a new event. We used purposive sampling to select institutions with relevant experience and knowledge to inform the study objectives. This enabled us to maximize the variation of institutions within our sample. We also used snowballing techniques by asking respondents for other potential informants. The study's population consisted of key players in COVID-19 vaccine distribution and community engagement in Mozambique.

The inclusion criteria for institutions were the following: have been part of the national committee and/or subcommittees for the introduction of the COVID-19 vaccine (planning and implementation subcommittee, logistics and cold chain subcommittee and advocacy, social mobilization and communication); other non-health governmental organizations; CSOs representing vaccine priority groups, women, the elderly, refugees, people with disabilities; and the private sector. For individuals, the criterion was to be the representative of the institution selected for the study or have been indicated for the interview by the representative of the institution selected for the study. We included institutions based in Maputo city because of their accessibility, except for one with representation in the city but headquarters outside it. In all, we contacted 31 institutions, out of which 20 responded. 19 institutions completed an interview, and one did not take part in the study because the project related to COVID-19 had ended and the team involved had been dismissed.

### 2.2 Data collection

We designed the interview guide according to the components of the COVID-19 response and piloted it by asking for an expert review. We improved the guide in terms of clarity and flow as we interviewed participants. To recruit participants, formal letters of invitation were sent to selected institutions/organizations inviting them to participate in the study. The interviews were conducted mostly in person, but also over the phone and on-line depending on the availability and preferences of interviewees. Interviews were conducted in Portuguese and lasted about one hour. The interviews were audio-recorded (with the written consent of the participant), transcribed verbatim, and anonymized for analysis. Participants were asked about their role in the organization and responsibility in the vaccine distribution and delivery in Mozambique, years of service in management or leadership position in the public health system, their opinion on what worked well with the country's COVID-19 response and what could be improved as preparation to future pandemics (especially about the part of the COVID-19 response implementation they were involved with). The interviewer was female and had been trained in qualitative methods. Data was collected in four months (July to October 2024). Ethical approval was obtained from the Institutional Review Board in Mozambique (IRB00002657) (Ministry of Health, approval No. 04/CNBS/24).

### 2.3 Data coding and analysis

Audio recordings were transcribed verbatim, anonymized for analysis, and coded using a computer-assisted qualitative data analysis software Maxqda 2020. Data analyzed using a deductive thematic approach, which is driven by the researcher's theoretical or analytic interest in the area, and is thus more explicitly analyst-driven (24). The following steps were followed: (i) read and familiarize with the data; (ii) generate code and respective list; (iii) code data using the code list previously generated, (iv) systematically review the codes generated and identifying the most significant and frequent codes that directly relate to the research question, (v) categorize codes into themes (vi) explore the relationships and connections between categories of codes and summarize data using data reduction tables and produce summary tables, (vii) interpret themes and write-up (25). Additionally, a validation meeting was held, in which research participants were asked to check the accuracy of the results and interpretations. Their feedback was used to validate, clarify and supplement the researchers' interpretations. The meeting was constructive as it helped to ensure trustworthiness and quality of study findings.

## 3 Results

In total, 19 participants representing various stakeholders were interviewed (Table 1), of which 16 were face-to-face, one online, one by telephone and one sent the response by email. Most participants were male (68%) and had between 11 and 20 years of experience in public health management and vaccines (Table 2). The institutions/organizations represented by the participants contributed to the response to COVID-19 with planning, logistics, human resources, equipment and materials, capacity building, risk communication, monitoring and evaluation, and impact mitigation (Table 3). Findings regarding what worked and what could be improved, which have been translated from Portuguese to English, were organized by area of response to COVID-19. These findings are explained in the section below and are supplemented by the participants’ quotes (Table 4).

**Table 1:** Number of participants and stakeholder representation.

Type of organization	Participant's organization	# of participants	In person interviewed	On-line interviewed	Over the phone interviewed	Emailed response
Non-health government organization	Gender, children and social action	1	x			
	Penitentiary services	1	x			
Health government organization	Epidemiological surveillance program	1	x			
	Implementation, management, regulation and supervision of health research activities	1		x		
	Extended Program on Immunization (EPI)	1	x			
	Health promotion program	1	x			
	Supply chain program	1				x
Multilateral organizations	Children's fund	1	x			
International health organizations	Clinical support	4	x			
Civil society organizations	Elderly association	1	x			
	Diabetics’ association	1	x			
	Refugee association	1			X	
	LGBTQ + association	1	x			
	Associations of people with disabilities	1	x			
	Intercollegiate religious platform	1	x			
Private sector	Information system	1	x			
TOTAL		19				

**Table 2:** Socio-demographic characteristics of participants.

Characteristics	#
Age	
25–45	9
46–65	7
66–70	1
No age provided	2
Gender of respondent	
Female	6
Male	13
Gender of interviewer	
Female	1
Male	0
Education	
PhD	5
Master	5
Licenciatura (intermediate degree between bachelor's and master's degree)	7
Bachelor	1
No education level provided	1
Occupation	
Executive director/country manager	6
Project director	1
Project assistant	1
CEO	1
Head of division/department	6
Immunization specialist	1
Researcher	1
Clinic director	1
Project manager	1
Years of service in vaccines	
0	5
1–5	4
6–10	3
11–20	4
21–25	2
Information not provided	1
Years of service in public health management	
0	7
1–5	3
6–10	
11–20	7
21–25	
Type of organization	
Health-related government	5
Non-health-related government	2
Health-related multinational	1
Health-related national	1
Health-related international	3
Private	1
Civil society	6

**Table 3:** Contribution of the organizations participating in the study to the response against COVID-19.

Contribution to the response against COVID-19	Organization					
	HRG	NHRG	HRI	HRM	PS	CS
Planning and monitoring						
Investigation of the outbreak and design of the first guidelines and control of the disease in the country	x					
Conducting epidemiological survey to identify the most affected populations in order to subsequently design the strategy for implementing the Covid-19 vaccine, based on priorities in terms of age groups, vulnerable populations, etc.	x					
Elaboration of the response plan, including the description and quantification of the target groups for receiving the vaccine	x		x			
Training, education, health communication (design of educational materials—posters, scripts for radio and TV spots)	x					
Mobilizing resources for vaccine distribution			x	x		
Financial resources provision			x	x	x	
Ensure the organization of planning meetings and respective follow-up			x			
Coordination and monitoring during the implementation of vaccination campaigns	x		x			
Design of vaccination information collection tools			x			
Establishing links between the health sector and the private sector for health promotion, including the distribution of vaccines			x			
Logistics						
Vaccines procurement	x			x		
Vaccine purchase	x				x	
Resource mobilization				x		
Vaccine administration	x					
Facilitate the conservation and distribution of vaccines [donated by different countries, vaccines provided through the COVAX mechanism (Global Access to Covid-19 Vaccines), and vaccines purchased by the government through bilateral agreements] from manufacturers to the last mile				x		
Financing outsourced transport from the provincial level to the last mile			x	x	x	
Provision expertise in the field of transport, supply chain, and the use of data					x	
Financing outsourced transport from the provincial level to the last mile			x	x	x	
Provision of fuel and means of transport for mobile vaccination brigades to travel to companies and other vaccination sites in the communities			x			
Provision of expertise in the field of transport, supply chain, and the use of data					x	
Support (e.g., stipend) for health professionals to travel to the districts to implement vaccination			x			
Provision of airtime to facilitate the communication and sending of data from the peripheries (health facilities), district headquarters, to the provincial level and from the provincial level to the central level			X			
Human resources						
Provision of human resources (e.g., typists) to assist in the typing of information at Ministry level, to facilitate the issuing vaccination certificates			X			
Equipment and material						
Provision of PPE (Personal Protective Equipment), masks, alcohol, etc.			X			
Capacity building						
Training of all technical staff (doctors, nurses, etc.)			X			
Risk communication						
Dissemination of information and mobilization on prevention measures, including vaccines, on radio, television, social networks and call centers, etc.	x	x	X	x	x	x
Ensure that people know where services are available and who is eligible	x		X			
Preparation of communication tools (preparation of leaflets, radio spots).	x		X			x
Create partnerships with different civil society groups such as religious leaders, influencers to support the campaign)	x		X			
Preparing messages on prevention measures including COVID-19 vaccines			X			x
Monitoring and evaluation						
Monitoring and evaluation			X			
Carry out evaluations to understand the perceptions of health workers and the community about the vaccine			X			
Impact mitigation						
Ensuring that some people in the community whose sources of income had been affected had at least enough food to get by during the critical period;						

HRG, health related government organization; NHRG, non-health related government organization; HRI, health-related international organization; HRM, health-related multinational organization; PS, private sector; CS, Civil Society Organization.

**Table 4:** What worked and what could be improved by response area.

Response area	What worked in the response to COVID-19?	What could be improved in preparation for the response to future pandemics?
Vaccines demand generation	Promotion by religious, community and political leaders, influencers and activists	Strengthen the dissemination of information
	Use of data on the needs and priorities of target groups to inform strategies	Use of inclusive approaches to provide information about prevention measures, including vaccination
	Use of a tracking system to monitor rumors about COVID-19 and respective vaccines	Increase the budget for communication on outbreaks and pandemics
	The creation of a communication package for use by religious leaders	Strengthen the production of evidence to support the creation of demand for vaccines
		Improve the skills of health professionals for health promotion.
	Provision of daily updates on the incidence and prevalence of COVID-19 infection in the country	
	The provision of vaccination certificates	
Vaccine distribution (Supply chain)	Decentralization of the distribution process and investments in infrastructure and equipment	Continue to invest in infrastructure, equipment and with the decentralization of the distribution process
Vaccine allocation	Careful consideration of priority groups by targeting the highest risk groups	Provide clear information on the vaccine allocation plan
Vaccine delivery	Innovation, flexibility and adaptability of the extended vaccination program towards the needs of the target groups	Capacity building for health professionals and provision of equipment
	The steep learning curve of health professionals	
	Capacity building	
	Mobilization of additional staff	The use of more effective strategies to reach vulnerable and hard-to-reach populations
	Use of existing vaccination delivery structure	
Surveillance	Use of community agents as the first line of surveillance	Setting up observation centers for infection suspects
	The use of the existing sentinel surveillance of flu-like syndromes system	Further scaling up, diversification (using more accurate and simplified testing technologies) and decentralization of testing capacity
	The use scale up of the diagnostic testing capacity	Improve community agent's surveillance capacity
	Establishment of public-private and public-public partnerships	Centralize the surveillance process management
	The use of pre- existing vaccines efficacy monitoring and adverse events	
	The use of a simple and up-to-date definition of a COVID-19 case	
COVID-19 immunization verification		Disaggregate data on vaccine coverage
Response coordination and leadership	Political engagement at the highest level	Develop a strategic plan, a corresponding budget and fund to respond to outbreaks and pandemics
	A clear plan, strategies and a multi-stakeholder team	Improve efficiency of the vaccine distribution and coordination process
		Support community-based organizations so these can better support the implementation of the response to pandemics
		Improve health authorities financial and technical accountability
		Ensure that vulnerable populations are represented in the decision-making process
Partnerships to overcome the challenges health system	Recognition by stakeholders that health promotion is the responsibility of all sectors of society	Health authorities must share needs in advance
	Provision of human, financial and technical resources by stakeholders	
	Flexibility of partners	
	Weekly virtual meetings between the technical coordination group	
	Monitoring of activities from the central to the provincial level	
	Duration of collaboration (around 10 years) between health implementing partners and health authorities	
	Teamwork	
	Joint planning, implementation and performance evaluation	
	Existence of communication mechanisms between the public and private sector	
Impact mitigation		Development of a data base on vulnerable populations
	Continued provision of essential services for other diseases	Provision of psychological support to those that may be in need

### 3.1 Vaccines demand creation

#### 3.1.1 What worked?

Most participants reported that the strategies used to create demand for vaccines and other preventive measures have been successful due to:

The contribution of religious, community and political leaders, influencers (musicians, artists) and activists from different CSOs in raising awareness about the benefits of vaccination. For instance, the health minister was the first person to be vaccinated in the country. Additionally, testimonies from people infected and sick with COVID-19 were used to raise awareness of the disease.

“In terms of strategy..for creating demand..we produced materials..broadcasted on radio stations..At community level, we have a structure called the health committee..made up of influential people in the community: the neighborhood secretary, the matron.., the most active teacher..These people… were trained and… spread messages in the community..When we started having [COVID-19] cases here in Mozambique, we also used these people as witnesses… to speak first-hand about the situation they were experiencing, so..people could realize that the situation was real…it existed and each of us had to act to prevent..contamination from happening at community level” [Health government organization representative].

Use of data on the needs and priorities of target groups to inform strategies aimed at raising interest in vaccines.

“So, we researched..[because] we… noticed insecurity on the part of some professionals..From this [research] we… created messages..to motivate, to demystify rumors that were… being spread among health professionals, because this was an important group..also a higher risk group. So, it was… important to ensure that this group could be..vaccinated..The same also happened with adolescents. In relation to the other target groups, we [also] had to carry out..an assessment, which was to understand the extent to which people were prepared or able to..receive vaccines” [Health government organization representative].

Use of a tracking system to monitor rumors about COVID-19 and respective vaccines.

“We had a group that monitored rumors and acted on them by sending corrective messages via the [MoH] website, [a] Facebook [page], call centers and [organized] press conferences. This group assessed the information that could lead people to distrust vaccines and then suggested corrections to these information” [International Health organization representative].

The creation of a communication package for use by religious leaders to ensure that they disseminate correct information in a coherent way. This package was composed of strategies, approaches, materials, and a monitoring and evaluation plan.

Provision of daily updates on the incidence, prevalence, and mortality due to COVID-19 infection in the country, i.e., the number of people infected, discharged, dead and vaccinated. These updates have helped to raise awareness of the seriousness of the disease and the need for vaccination. Further, the provision of information on the advantages of vaccines (feasibility, practicability, and effectiveness) compared to non-pharmaceutical interventions (such as isolation, masks, closing public places, limiting gatherings, etc.) has also contributed to people's interest in vaccines.

“There was..clear communication …. every day..through social media…to inform us how many people were infected, how many people had been discharged, how many people [had] died..and this attracted attention, because in a way, people became more aware..[International health organization representative].

The provision of vaccination certificates

“In any border you couldn't pass..without this document [vaccination certificate]..You had to have this document..otherwise you couldn't pass..After having been vaccinated you could pass…Therefore, for me this was positive and..very important” [CSO representative].

#### 3.1.2 What could be improved?

Many participants recommended strengthening the dissemination of information to ensure that all segments of the population, in urban and rural areas, receive information about the need for vaccines and their respective effectiveness. This could be done by providing correct and coherent information, in a way that is easy to understand and using several approaches, including the use of structures within existing health programs (for example, the use of CHWs in the HIV program) and other government sectors, such as the Ministry of the Interior and Education.

“From a human rights perspective, we cannot force people..to adhere to vaccines or tests. What we must do is ensure that people have the right information, know the advantages and disadvantages of making the decision they need to make..What can be strengthened? We need to be assertive in designing the messages that we want to..get across at community level, so that..there is no room for manipulation due to the speculations that people hear out there. The speeches that..discourage people…to adhere to both the tests and the vaccines..only gain ground when there is no consistency in the … information that we pass on..Therefore, when we pass on correct and coherent information, clearly, people..aren't going to fall for..misinformation” [CSO representative].

Some participants recommended the use of inclusive approaches to provide information about prevention measures, including vaccination, i.e., sign language, local languages and in some cases foreign languages. Additionally, those chosen to share information should be trained on the outbreak and to be aware of local language variations and cultural issues. Portuguese is the official language in Mozambique, and it is spoken by 37.7% of the population. There are 19 national languages (and 43 dialects), including Makwa, Swahili, Yao, Nyanja, Chuabo, Ndau, Sena, Bitonga, Changana, and Ronga (22).

“Some [refugee] communities..did not have people capable of..transmitting..the messages [for them], for instance, the Somali community..In the event [of a future pandemic]..we could improve a lot..We should train…, equip the people …who are going to provide information..in other languages..[to] have a basic understanding of the disease..What we saw was that people were translating according to their knowledge of the language but not necessarily [about] the disease..[Also], when translating..messages…it could be done comprehensively, [i.e.,] not only [with] the refugee community [in mind], but also [the local communities]..Just to give you an idea.., the Makwa language … has dialects…which means that when you translate it, you must take [its variants] into account. It is hard work, but to save a life we should never..runaway..from work..” [CSO representative].

Some participants recommended increasing the budget for communication on outbreaks and pandemics.

“Well..it is what I keep saying [communication] is important but..the budget allocated to communication is exceedingly small. Communication..is important because it is a..cross-cutting issue, it is in all areas. For example, for the vaccine to reach the population and be used there must be [communication]..The budget is extremely limited, which limits us from implementing the necessary interventions… and to be able to improve acceptability. Therefore, I..think that allocating resources to the area of communication should be a must..” [CSO representative].

Some participants recommended the production of evidence on an ongoing basis to inform communication strategies, by setting up an observatory that would be ready to conduct assessments of people's expectations of outbreaks whenever necessary. Produce evidence on an ongoing basis to inform communication strategies.

“..When vaccines were..introduced, we did..a quick study..which helped us to target our messages and be able to respond to what..people's expectations were. In this area..we need to explore more, because it [research] was done sporadically..but maybe in the future..we could have, … a kind of observatory, a specific area that looks at this, right? Which I think would be worthwhile..so that, when we have an event [outbreak/pandemic], we [could] count on this organization to be able to do these surveys…It could help feed our communication strategies …” [Health government organization representative].

Some participants recommended improving the skills of health professionals for health promotion.

“Well, we learn by doing..What I feel is that we need to specialize..in this component of public health emergencies… [i.e.] the communication component..It's something that we..need to learn about with a bit more of science..… We already know that we are susceptible to public health events, so training at national, central, provincial and district level..is essential, without a doubt” [Health government organization representative].

### 3.2 Vaccine distribution (the supply chain)

#### 3.2.1 Mozambique's vaccine distribution process

Historically, the supply chain for routine vaccination has followed a multi-tiered system. The manufacturers send the vaccines to a national warehouse in the capital (Maputo), which sends quarterly shipments of vaccines to most provincial shops by plane and trucks. The health facilities (last mile) order vaccines from the districts monthly and, although national policy dictates that the district warehouses deliver vaccines to the health facilities by truck or motorbike, many health facilities must send health workers by public transport to collect the vaccines due to the unavailability of vehicles or the lack of funds for fuel in the districts. The *ad hoc* nature of transport contributes to delays in sending vaccines to health facilities (26).

#### 3.2.2 What worked?

According to some participants, vaccine distribution for COVID-19 followed the same process as routine vaccination, except that COVID-19 vaccines were not sent quarterly to the districts, but as soon as they arrived in the capital city, and mostly overland. This was so because of the high demand for vaccines and their expiry date.

Investments have been made in the supply chain to improve vaccine distribution, namely: purchase of 20 ultra-cold chain equipment; increased capacity nationwide in terms of space (rooms) to install ultra-cold equipment at national level; purchase of material such as isothermal boxes and fridges (for about 90% of all health facilities in the country); purchase of vehicles to support the transportation of vaccines; the purchase of motorcycles to help access areas that are difficult to reach; and renting and construction of warehouses and capacity building.

**“**We invested in [ultra-] cold chain equipment. We did not have this type of [equipment]..in the country. We had support for the acquisition of twenty pieces of ultra-cold chain equipment. We also made..other investments..[i.e.] the..vaccination itself..[which] was supported both by funds from the Ministry of Health and from partners, enabling that whole vaccination movement to the communities..We purchased..material: isothermal boxes [and] coolers. The room that houses the ultra-cold chain[equipment], was also part of the Covid-19 vaccination investment. We also had support in acquiring vehicles to support the transportation of vaccines…[Health government organization representative].

Acceptance of all vaccines that were pre-qualified by the WHO and the country's regulatory authority i.e., AstraZeneca, Sinopharm, Johnson and Johnson and Pfizer. The fact that the country did not have preferences for specific vaccines, helped secure sufficient vaccines.

#### 3.2.3 What could be improved?

Some participants reported that to reduce costs, stopping points and allow for greater fluidity and speed in the delivery of vaccines to health facilities, the efforts to decentralize the vaccine distribution process must continue, i.e., vaccines must be delivered to the regional warehouses (south, center and north) and, from there, these must be channeled to the intermediate warehouses in districts in all provinces. From there, these must be distributed to health facilities. Additionally, the following actions should be considered:

(i) Speed up the customs clearance of vaccines.
(ii) Use air transport from the provincial capitals to the districts
(iii) Use drones
(iv) Use logistic information systems
(v) Increase the infrastructure of the ultra-cold chain at regional and provincial levels.
(vi) Control temperature remotely

“We identified the need to strengthen the distribution chain itself. We learned that..getting all the products from Maputo [the capital in the south of the country] and then distributing them to Cabo Delgado [a province in the north of the country] is very costly..[During COVID-19], we sent vaccines to provincial headquarters, and from there to district headquarters, and from there to health facilities. The strategic pharmaceutical logistics plan..states that the products must arrive in three different regions [south, center and north]..What do we envision? That in next pandemics or if we must receive vaccines in large numbers..we do not only receive them in Maputo..[but] in each region..directly, to allow distribution to flow more smoothly..and facilitate..a timely response. We have made progress with the investment because we already have warehouses operating..in the three regions..The same plan also includes setting up intermediate warehouses in all the provinces..What will these warehouses allow? Each warehouse will supply a certain number of health facilities directly..to capitalize on resources, reduce stopping points and allow greater fluidity so that delivery to the health facilities is quicker enabling a rapid response” [Health government organization representative]**.**

### 3.3 Vaccine allocation

#### 3.3.1 What worked?

Most participants reported that the phased allocation of vaccines, which prioritized groups that were most likely to infection, serious illness and death from COVID-19, resulted in an equitable and effective vaccine allocation. Since vaccine doses were not available at required quantities at once, the Extended Program on Immunization (EPI) had to be flexible and strategic in allocating them to the population. The EPI is responsible for vaccination in the country. It was launched in 1979, after the first vaccination campaign following national independence (27). The EPI prioritized the population according to epidemiological profile, permanence in places or environments where the implementation of prevention measures was difficult, vulnerability to serious illness, and logistical and operational aspects, i.e., people most likely to contract and/or die from COVID-19 (health workers, the elderly, people with chronic illnesses, the defense forces and prisoners). Refugees, adults, and adolescents received the vaccines in subsequent phases. Lessons from COVID-19 on prioritization and quantification are now applied to routine vaccination.

“I think..the vaccination program has learned to be flexible…especially when it came to distributing vaccines… There was a plan that said: we must vaccinate ×  number of people, we need ×  doses, but these doses didn't arrive in the country at the same time..Thus, one of the lessons that the vaccination program has learned, which I..congratulate, is on how to phase the [distribution] process. If it is not possible to have all the vaccines at once, how can we prioritize? So, one group was prioritized when the vaccines arrived…later … there was an opening for other target groups, when there was a larger quantity of vaccines..One of the lessons from this is that recently..the vaccination program was, for example,..doing an exercise to improve the quantification of vaccines for routine..and there are methodologies that they've applied that show that this flexibility is being adopted..it's a learning from Covid[-19]..In the future if an emergency arises, we're better prepared” [International health organization representative].

#### 3.3.2 What could be improved?

Some participants recommended providing clear information about the vaccine distribution plan, to reassure the population that the vaccine will reach everyone.

“When the vaccine came, it was for priority people..e.g., the elderly..and the others asked why not us? We also want to be vaccinated. So, there was a lot of unrest..in the community. I remember..when the vaccine was introduced… [I] had family members..elderly people. We queued at the central hospital so they could receive the vaccine. It was a mess, a real mess.., there were queues..people fighting, [saying] we also want it [the vaccine]. So..there should have been a bit more work in ensuring..that everyone would be vaccinated,..[because] there was a lot of concern at the time..that the vaccine wouldn't be enough for everyone” [CSO representative].

### 3.4 Vaccine delivery

#### 3.4.1 What worked?

Some participants mentioned innovation, flexibility, and adaptability of the EPI to meet the needs of the target groups, as determining factors for the successful administration of vaccines. In phases one and two, which targeted health professionals, the elderly people living in nursing homes and nursing home workers, and people with chronic conditions, vaccination was conducted in health centers, district health directorates, nursing homes and associations, for example, the elderly and diabetes patients. In phases three and four, vaccination was extended to communities (e.g., businesses, gardens, schools) and centers of concentration/places of agglomeration (transport terminals, etc.) to achieve two objectives: to reach a greater number of people and not to overcrowd health facilities. Additionally, the flexibility and the steep learning curve of health professionals in dealing with diverse types of vaccines (AstraZeneca, Sinopharm, Johnson and Johnson, and Pfizer) in a short time, also contributed for the successful administration of vaccines.

“The vaccination program managed to achieve its vaccination targets. Part of the target group in the first phase was… elderly people..who lived in nursing homes. So..the team went to these nursing homes..and managed to reach this target group. Therefore, [the] strategy was successful, because the vaccination program adapted to the needs of that target group…Health professionals were given the vaccine at the health center or at the district headquarters, where it's usually much easier for [them] to go and get the vaccine. So… [the strategy] has been successful in this sense… In the second phase of vaccination, when the country begun to receive a slightly larger quantity of vaccines, it [the program] realized that just offering the vaccine at the health center would probably not be enough..therefore, concentration points were created, for example, football pitches..places of agglomeration..public transport..terminals…” [International health organization representative].

Some participants reported that the use of pre-existing vaccination delivery structure made up of health facilities, pre-defined vaccination places in the communities and respective community leaders was crucial to mobilize people for vaccination.

“The strategies [used to administer the COVID-19 vaccine] have been successful because Mozambique's health structure favors it. At provincial level, we have..the peripheral health facilities and fortunately we already have..some..pre-defined vaccination places [in the communities]..We also have régulos [community leaders], who are already..familiar with the vaccination program that is implemented at country level..[and] they can easily mobilize the population..to join the vaccination campaigns in a short notice. We have another strategy at national level, which is the [use of]..APEs [community health workers (CHWs)]. These are individuals who are part of the national system who are already..within the communities…Therefore, this [structure] helps in terms of..preparation..as well as mobilization for..vaccination” [International health organization representative].

#### 3.4.2 Public-public partnerships

Some participants reported that the mobilization of additional staff, such as nursing students, health professionals who were not normally dedicated to vaccination, pensioners and non-technical staff, helped in overcoming the challenge related to the lack of manpower for the COVID-19 vaccine delivery. Additionally, the support of the national defense forces was also key for vaccine delivery in Cabo Delgado (a province in the north of the country) given the situation of the armed conflict.

“To meet the high demand [for vaccines]..students..from the health institutes have been involved…Other health professionals who don't normally dedicate themselves to vaccination were [also] mobilized” [CSO representative].

#### 3.4.3 What could be improved?

Many participants recommended the use of more effective strategies to reach vulnerable and hard-to-reach populations. One of the main limitations in distributing COVID-19 vaccines was reaching people in rural areas, reaching some elderly people who were not in nursing homes and could not travel to vaccination sites; reaching people with disabilities and the LGBTQ community who often suffer discrimination by some healthcare providers. A minority of participants said that men and pregnant women were also less likely to be vaccinated, but for different reasons, the latter being due to a lack of guidance on the safety of vaccines for them. Participants recommended strategies such as the use of geospatial systems, the use of mobile and door-to-door brigades as a way of reaching these populations.

“I think that people with disabilities were the least reached..Starting with communication, which was not inclusive..[Additionally] vaccination places were not accessible…I'm a person with a disability..I got the vaccine, but it was with a lot of sacrifice…I got the vaccine at a school..and I had to climb stairs..I had to climb stairs, imagine… Ok, I went up, very well. Although [I went up] with a bit of sacrifice.., with a lot of effort, I managed to reach upstairs. Now imagine going down the stairs after being vaccinated with crutches. It was difficult…Therefore, it [the vaccination] was..not a very inclusive process, although, after a lot of noise on social networks..the Ministry of Health (MoH) improved..so much so that in the President's latest reports [communication to the nation]..we could see the interpreter there..using sign language to facilitate communication”[CSO representative].

### 3.5 Surveillance

#### 3.5.1 What worked?

Some participants reported that the use of the pre-existing sentinel surveillance of flu-like syndromes system and vaccine adverse events facilitated the early detection of COVID-19 cases and allowed for the consequent tracing of the respective contacts. Regarding the monitoring of adverse events, it has been strengthened by the creation of surveillance committees based in the districts. These committees shared information regarding adverse events with the provinces and these shared with the World Health Organization (WHO) office in the country and from there it was shared with WHO headquarters. Concerning vaccines efficacy, it has been monitored by cross-referencing the patient's condition and the type of vaccine received. This control has been effective in showing that, regardless of the type of vaccine received, most serious cases of the disease occurred in unvaccinated people. Moreover, investments have been done in health communication system, particularly on geospatial systems, to monitor vaccine efficacy by mapping places with high infection rates and prioritize them for vaccination; places with high vaccination coverage and compare their vaccination coverage with their infection rate. However, this geospatial system has not been used during COVID-19, but it is being currently used for routine vaccination.

“[surveillance] was very successful.., the lethality rate, the morbidity rate, in our country [was] extremely lower when compared to some countries in the region or even in the world. We [Health authorities] have already implemented sentinel surveillance of..flu-like syndromes. … it was assertive… in that we were able to detect the first case early on, and whenever we had a case..detected in the health facilities..it was easy to do contact tracing..We were prepared.., we knew that..viral, flu-like syndromes have a high propagation power, so we had activities that allowed us to be prepared for..things like that [COVID-19]” [Health government organization representative].

Most participants mentioned that at the start of the pandemic, the country did not have sufficient testing capacity. Samples were taken and sent to the National Health Institute in the capital. This was a challenge, especially for the provinces. However, capacity was built up over the course of the pandemic and the scale up of the diagnostic testing capacity allowed cases to be detected at various points such as borders, airports, and health facilities. Participants also said that the increase in screening capacity had also helped to raise awareness of flu syndromes in general.

“[The testing strategies]..have been extremely successful..It was possible to expand, for example, laboratory testing capacity, molecular biology to every province in the country, to expand rapid testing and to sensitize the public to the need for testing..It was not common for..people to be tested for flu syndrome[s]. With the laboratory testing strategy, the massification [and] the dissemination of information (the testing for flu syndromes) was also..achieved” [Health government organization representative].

Some participants said that public-private and public-public partnerships were key to meeting the demand for tests and to sharing learning and good practices.

“Another approach..was the use of laboratories..of [universities and research centers]..This synergy of using the network of laboratories available in the country..both the public and the private network, was useful. We used..the private network to conduct tests. There was interoperability between the systems so that we could have data..from these private laboratories. It was a success story..as a country, of using all the available resources we had access to..without restrictions, without concerns about cost, without looking at issues related to individualized interests. The issue was treated as a problem of the nation, that needed solutions..we created the synergies for this purpose, I think it was a success story..” [Health government organization representative].

Some participants reported that the use of a simple and up-to-date definition of a COVID-19 case allowed all stakeholders, especially CHWs, to understand how to suspect that a person had COVID-19, which enabled the mass identification of cases and their referral to health facilities for screening.

“..looking at the case definition.., we have always tried to use simple, understandable language..so that ..any health technician in the clinical area would be able to understand..and that has helped a lot in identifying cases… We've always been guided by having a community case definition, which is to ensure that community actors …made a good referral of patients to the health facilities..and that our colleagues in the health facilities, using the classic case definition, would be able to screen [for COVID-19]” [Health government organization representative]**.**

#### 3.5.2 What could be improved?

Some participants recommended setting up observation centers for infection suspects. These centers should also be set in prisons (which also lack this infrastructure) and should have minimum accommodation and food conditions to be attractive. In addition, the participants welcome the creation of the emergency committee, which could enable a multi-sectoral response to isolate and treat cases, should the need arise in the future.

“A fundamental issue..in next eventualities: we must understand the origin of the cases..and the requirement to isolate cases..There was a great limitation in terms of how to isolate someone who comes with COVID-19 symptoms….[and]..the mechanisms for monitoring this measure..weren't, very clear..So, once again, the need for an..inter-institutional..response, to allow various players..to share [responsibilities] in this matter of isolating and treating cases. There is a need to reinforce..capacity in terms of response..immediately after the first cases are detected..The national public health directorate created..the..Emergency Committee, which includes various institutions, and … is where we'll be able to call on the..multisectoral response..and the office is already working, and has a plan..which describes the role of each institution in the event of..future pandemics” [Health government organization representative].

Many participants recommended further scaling up, diversification (using more accurate and simplified testing technologies) and decentralization of testing capacity, as this would strengthen the surveillance system to become active, permanent, accessible, sensitive, and equitable. Additionally, they also recommended the quantity of human resources, equipment, and transport for the implementation of epidemiological surveillance activities to be increased.

“The surveillance of vaccine-preventable diseases must be..permanent..[it] must be equitable, because the disease does not have..a choice of location..of person, of sex. There was more availability of disease surveillance in urban centers than..in rural areas. [Also], when COVID [-19] started, it was not..cheap to get tested, you had to..pay..Therefore, this..does not bring equity, why? Because I can be ill and have no money, I have no way of knowing if this is COVID [-19] or not..Thus, surveillance of..vaccine-preventable diseases must be..accessible, sensitive to detect whether we have it or not” [International health organization representative].

Some participants recommended strengthening the capacity of CHWs to better identify diseases, know how to report them to health facilities and know which control measures to apply at community level to prevent the spread of diseases.

“It is particularly important that we equip our agents [CHWs]…give them practical instructions on how to identify diseases, how to report..them to the health facilities..and..[how to] control [and] prevent their spread at community level. That is the part we need to strengthen as a system: community surveillance… because the intra-hospital one is very well established..” [Health government organization representative].

Some participants recommended centralizing the surveillance process management, by designing systems that allow information to come from different sources and arrive in an integrated, unified and standardized way at an institution, preferably a public one. This institution would control the entire data management process, using a tool that records, for example, data from the request of a test, the issuing of the result, to the verification and follow-up of the suspected case and respective contacts. In addition, participants recommended the use of early warning systems for outbreaks, using historical data.

“..one of the challenges was that this information [COVID-19 test results] was kept in the various locations..It was a challenge because there was no..platform, agreement,..rule, decree,..that said [that] all COVID [-19] information..had to be centralized..The idea is that..any clinic could [collect] the information, but at the end of the day, the information should be sent the same place..For example, in other countries, this was an entity that was created by the state to manage [the process]…There were public and … private laboratories..[all] carried out tests… issued [vaccination] certificates and everything, but the information was managed in a single platform..so the sharing [of] information already existed by default” [Private institution representative].

### 3.6 Vaccination verification

#### 3.6.1 What could be improved?

Some participants recommended the disaggregation of data to facilitate knowledge of the level of vaccination coverage of the various groups. Additionally, the use of geospatial systems can help to understand how each spot contributes to vaccination coverage.

“..maybe it [data] is [currently] not a level of disaggregation that allows you to do that [to know which groups have been less reached by vaccination]..These [figures] don't show much, because when you look..in aggregate for the province, you notice that we practically have a coverage of more than 90%, in almost all the provinces…[However], there are probably some places where the target group may have been underestimated..But the figures don't necessarily show that…Often, when there are coverage difficulties..when you can't have a very strong level of disaggregation, it's difficult to know which areas haven't been reached..” [International health organization representative]**.**

### 3.7 Planning, coordination and leadership

#### 3.7.1 What worked?

Participants were unanimous in stating that the coordination of the response to COVID-19 has been successful, due to the following:

Political engagement at the highest level. The commitment of the president, governors, ministers and secretaries of state in the response to COVID-19, to ensure the supply of vaccines and promote their acceptance, sent a clear signal to the population about the threat that the pandemic represented.

“We joined the COVAX [COVID-19 Vaccines Global Access] mechanism, which was..a commitment from the highest figure in the country..I'm talking about the President of the Republic..He really understood that it was important for the country to join the COVAX mechanism to be able to do mass vaccination. If you do a bit of research on the Internet, you'll see that at a regional level, even [among] some countries on other continents, Mozambique had the best vaccination coverage..but it was really a question of commitment. …The involvement..[of] the political leader at the highest level automatically [influenced] the provincial [leaders i.e.] the Secretary of State, the Governors…” [Health government organization representative].

A clear plan, strategies and a multi-stakeholder team, which facilitated the collaboration of traditional and new partners, i.e., health national and international implementing partners, non-health public institutions, private institutions and CSOs. Additionally, Mozambique was on the list of the first 10 countries to draw up a COVID-19 vaccination plan in Africa.

“..when there is a clear plan, a clear strategy about what needs to be done, funders are willing to come and help..the MoH. Because..it [MoH] had a strategy, a..very good vaccination plan against COVID-19..[it] was easily able to mobilize..partners [and] COVID-19 vaccines. So… that's important..” [International health organization representative].

#### 3.7.2 What could be improved?

Many participants recommended the development of a strategic plan, with a budget and a fund to respond to outbreaks and pandemics, which should be approved at the highest political level and reviewed every five years. The plan should be accompanied by a communication and monitoring plan and a coordinating council.

“..Another thing..we also need to have funds for pandemics…For example…the Global Fund..[is a] funding [for] malaria, HIV and tuberculosis, right?..We have large funds for these areas, why not for pandemics? Let's open a specific fund for pandemics in the country, because outbreaks…pandemics happen. So, it [pandemic] should also be on the government's agenda as a public health problem, a problem that needs attention” [CSO representative].

Some participants recommended that the multidisciplinary Technical-Scientific Commission must recognize and respect existing expertise and always base its decision on evidence. Some of its decisions were politically based and this created bottlenecks for the COVID-19 response. Participants also recommended improvements in the efficiency of the coordination of the vaccine distribution process to avoid wasting resources such as money and time. Furthermore, they recognize that the inclusion of different and varied groups in the planning of health interventions to respond to emergencies is fundamental, as it allows for the sharing of knowledge and experiences, thus enriching coordination. However, some aspects such as the provisions on the size of the technical group, the duration and the means used to hold the meeting need to be weighed up. In addition, they also recommend **the Ministry of Health to create a programmatic platform to visualize what is happening in terms of funding**. What happened is that, while the MoH should have been the coordinator of the funds intended to respond to COVID-19, it was not aware of the existence of certain funds, especially those received by implementing partners at provincial level. The consequence was a lack of visibility of how much was available for the COVID-19 response and inefficient use of funds as it led to duplication of activities, failure to prioritize activities and to monitor how the funds were used.

“..I don't think [the leadership] has always been..assertive..There were a lot of problems with the quantity of vaccines..The leadership, in a way..put pressure on the vaccines to be distributed as soon as they arrived..[without taking into consideration] the costs or the planning..Therefore..in that regard, the leadership didn't [succeed]. But from the point of view of..leading everyone involved, it went very well..but for those small aspects where..the vaccines were within the expiry date, we could have waited so that the delivery could have been better planned..” [International health organization representative].

Some participants recommended supporting CBOs so that they can better support the implementation of the response to pandemics.

“..the leadership was good. Now, what can be improved is: as well as giving direct support,..the leadership must think about … how to channel..some support, [which] can be material..financial….so that they [CBOs] too [can] intervene directly in implementation..” [CSO representative].

Some participants recommended improvements in financial and technical accountability for the implementation of the response. There was a justified and unjustified misapplication of funds; and non-transparency in the use of funds allocated to the COVID-19 response, which even resulted in criminal prosecution in some cases.

“..I think what must be well..managed is..accountability..in the purchase of vaccines..both the technical and financial… is one of the things that can be improved in the future” [CSO representative].

Some participants recommended that CSOs and particularly those representing vulnerable populations must be involved in the design, planning and implementation of disease outbreak response. For example, people with disabilities and the LGBTQ community were not involved in the planning and implementation of the COVID-19 response. The EPI and the Global Alliance for Vaccines and Immunization (GAVI) counts with the civil society, as a co-partner in vaccination efforts and this must be brought to fruition.

“..I can even say that..at some point we were forgotten..they have forgotten about the organisations that represent people with disabilities, just as they have forgotten about people with disabilities themselves..To say that..people with disability were not involved in the process of vaccination against COVID [-19]” [CSO representative].

### 3.8 Partnerships to overcome health system challenges

#### 3.8.1 What worked?

Participants were unanimous about the importance and success of partnerships in overcoming the challenges faced in implementing the various components of the response against COVID-19. They mentioned that there was a certain recognition by stakeholders that health promotion is the responsibility of all sectors of society, which facilitated collaboration and promoted involvement of other sectors, such as the education sector, religious and community leaders, and the private sector.

“[The] health sector should not feel alone… It is responsible for promoting health, but it must involve others..The private sector has shown that it can buy vaccines..if it can buy vaccines, it can do other health promotion [activities] for its..employees..In the end it's..[a] question of..partnership with the private sector…” [International health organization representative].

Provision of vaccines; and human, financial and technical resources. The private sector contributed with about 10% of vaccines and this helped the country to vaccinate many people in a short time. Together with other stakeholders, the private sector also contributed with data, financial resources, a varied set of skills, backgrounds, and experiences beyond the healthcare sector. Please also see Table 3.

“..[partners contribution]..allowed us to speed up..the process..of making vaccines available at various levels. As you know, our country is vast, and getting the vaccines to every corner of the country is not an easy activity..But with the support of the partners, we were able to get the vaccines into all provinces..[within] five days of arrival in Maputo. So, this..speed was really thanks to their support..” [Health government organization representative].

Some participants mentioned the following aspects as having contributed to good collaboration between partners: Flexibility of partners; weekly virtual meetings of the technical coordination group; monitoring of activities from the central to the provincial level; duration of collaboration with the MoH (around 10 years or more), which facilitated communication, agreement on implementation and coordination; teamwork and joint planning, implementation and performance evaluation; existence of communication mechanisms between the public and private sectors, i.e., the public health law which made it compulsory for both parties (public and private sectors) to share data.

#### 3.8.2 What could be improved?

Some participants recommended health authorities to share needs well in advance, so that partners can better respond.

“I'm saying that sometimes..we're taken by surprise: we need this and that, but we're also a partner dependent on other [funders]..So, this component of..[requesting well in advance] for the partner..to be able to respond to certain requests needs to be improved” [International health organization representative].

### 3.9 Mitigating impact

#### 3.9.1 What worked?

Some participants mentioned that although the focus was on COVID-19, services for other diseases continued to be provided and this helped to mitigate impact. The prevention campaign slogan used “protect yourself”, helped to reinforce prevention and encouraged health seeking behavior. The set up of toll-free telephone call centers, through which people could call and ask for assistance was also useful.

**“**I think the country has learnt..as the campaign progressed, how to manage messages…We moved away… from “stay at home” [to “protect yourself”]. At some point, we realized that telling people to stay at home..was linked to economic losses..security or public health. We understood that, by telling mothers to stay at home, they ended up not taking their children for routine vaccinations…Therefore, there has been a transformation of the message to say: you can leave the house..if the problem you have or the issue you want to resolve outside the home … requires..being there in person..[but] go protected..There was a significant difference between saying “stay at home” and ‘protect yourself’..So “protect yourself” was very important..I remember the lesson of HIV-AIDS..we went from “AIDS kills” to “protect yourself”..Therefore,..the country has learnt overtime…[that] keeping people at home has had some damage..Moving from “stay at home” to..“protect yourself”, … was an important..restoration…” [International health organization representative].

Some participants said that the government had achieved its goal of helping more than a million people by making financial resources available as a way of mitigating the impact of COVID-19.

“We [the government] have managed to mobilize as many resources as possible to mitigate the impact of COVID-19. From what was forecasted at the time, which was to assist more than a million people, the sector managed to achieve that target. We have had support from..various international organizations for the implementation of actions related to COVID-19, [i.e.] monetary transfers, for six months to people who have been affected. So..the sector mobilized and supported” [Non-health government organization representative].

#### 3.9.2 What could be improved?

Some participants recommended providing psychological support if in the future there is a need to isolate people.

“I don't think financial support is enough, there is also a need for some psychological support. A person who is in isolation, the name says it, is in isolation. It means that they are cut off from interaction with other people in society..It can have a double impact on the person…, because they're ill, and then, as if that weren't enough, they're prevented from interacting with everyone else. So, it can affect them emotionally, psychologically..Therefore, … [it is] also very important to provide emotional and psychological support, so that the person obviously knows how to deal with that phase or that situation s/he is in..” [Non-health government organization representative].

Some participants recommended creating a database on vulnerable populations to quickly find them during pandemics.

“Well..you know, when we talk about..pandemics, these are things that happen..they are not planned..which means we must adjust our plans obviously…Therefore..we need..to have..a database of people who might need help, so that when an emergency occurs, we already know [who to look for]..so that we don't waste time looking for whom to help while the partner has already provided the money..we must do better so that in the future, when similar situations occur, we don't have these problems..” [Non-health government organization representative].

#### 3.9.3 Moving forward: lessons learnt in responding to COVID-19 pandemic

Lesson 1: Quickly agreeing on whether the country faces a health emergency is crucial for a timely and effective response.

“..what did Mozambique do that some countries couldn't do? Reach a consensus that COVID-19 was a threat..The President never appeared on TV to minimise the threat of COVID-19. Therefore… reaching a consensus [as a nation] is important..when you want to respond to an emergency. It is a lesson..[and] it is important during pandemics..A consensus on what is… affecting us..Is it a pandemic or not? Some countries are still debating whether..COVID-19 is a pandemic or not..in Mozambique that wasn't a debate..” [International health organization representative].

Lesson 2: In emergency situations, political commitment, leadership and the use of science-based interventions are crucial.

“We've had other major public health events, but we haven't had this kind of leadership involvement like we had with the COVID-19 pandemic. It's just a question of maintaining the structure and documenting it to serve as a lesson for future generations.., [for them to] see that this generation managed the pandemic in this way, [and that] if [they do the same], it can [also] work. I think the machine was good, because we had..the Technical-Scientific Commission..the health sector, other related areas..This model is perfect..If you look at it, we as a country did not suffer that much.., just do a basic search around the region,..It was a real success story, the response to the pandemic” [Non-health government organization representative].

Lesson 3*:* The government must be the one to lead the response to health emergencies.

“When we're in emergency situations, we have a lot of partners at the table..The lesson we've learnt is that the government must… have a plan [and] a budget.., [and] everyone must follow that plan..because if partners all start pulling in their own direction, everything gets out of control..It was a pandemic, and the partners were able to understand that, and the government took the lead.” [Multilateral organization representative].

Lesson 4: Public-private and public-public partnerships are essential in responding to health emergencies.

“This [COVID-19] was a problem..that affected everyone; [therefore,] it was not just the MoH..that had to deal with it…Government bodies create policies..civil society must be able to..implement..them..So, everyone in their sphere of action..must be able to monitor..respond or..help..so as not to..increase the number of victims..Our participation..in the private sector or..the ‘non-governmental organizations’ is to help the state implement them [policies]…” [Private organization representative].

Lesson 5: Prevention must be carried out at all levels: individual, family, community and institutional.

“[On this] issue of prevention..what we expect is that every person living in Mozambique becomes a health promoter..It's everyone's duty to do this. If a member of my family falls ill,..I won't be able to work, if it's a child they won't be able to go to school, they'll miss lessons, I mean, it's a loss for the family..Therefore, we must see this as something that each of us can do, we must collaborate, participate, get involved, be proactive in this process of educating … starting in the family circle..and going..upwards..to other levels” [Health government organization representative].

Lesson 6: Community health care workers must be the first line of surveillance.

“One of the things we've learnt from COVID-19 is that surveillance must start from..the community..And..for that, the MoH, with its partners..began to introduce, to train the CHWs..to be the first line of surveillance, because they are the ones in direct contact with the population… and they are trained for that. … Therefore, the involvement of the community is important” [Multilateral organization representative].

Lesson 7: For maximum effect, it is necessary to combine several communication approaches.

“Another lesson that..was worth learning..has to do with..combining several communication approaches, not looking at just one..but at all that are available. We use community actors at community level, but we also have the radio, so if we combine them …This person hears this message repeatedly on these different channels..then at the end of the day..s/he's for sure going to change his/her behavior, isn't s/he? There's also the religious leader, the community leader, who also spreads the message as reinforcement. Therefore, it's important to combine all these communication strategies, so that at the end of the day, we can..achieve what are..the results or objectives” [Health government organization representative].

Lesson 8. Vaccine donations must be predictable and reliable.

“Vaccine donations must be made in such a way as to allow countries to plan their receipt and implementation of vaccination activities to best achieve the targeted coverage rates” [Multilateral organization representative].

Lesson 9: It is possible to implement and sustain public health measures in Mozambique.

“One of the lessons learnt is that, at the end of the day, it is possible to establish public health measures in this country: to make sure that schools have water, that..there is sanitation in the environment, and everything else..These things should not be relaxed while waiting for another pandemic…” [International health organization representative].

Lesson 10. Document the lessons learnt in responding to pandemics.

“We must have the practice of documenting lessons learnt..in a quick document, an A5. A small, flexible, quick document..that people can refer to..because generations pass..don't they? A small manual, where one can consult..[and say] at the time of 2020..we had COVID [-19] in Mozambique. How was the country prepared? How was the implementation in the laboratory component? In the [vaccination] promotion component? In the surveillance component? How did the country react to it? Therefore, a brochure, something written..with lessons..would be extremely important for the country..” [Health government organization representative].

## 4 Discussion

According to our participants the response to COVID-19 in Mozambique, in particular the distribution and delivery of vaccines, was successful. The vaccination program vaccinated 97% of the target population and 68% of the general population with the primary dose (one dose for Johnson and Johnson vaccine and the first two doses for all other vaccines administered in Mozambique). Key drivers of the successful response were strong leadership; a clear plan and strategies; a functional coordination mechanism; the use of evidence to make decisions; a phased allocation of vaccines, which prioritized groups that were most likely to infection, serious illness and death from COVID-19; investments in capacity building, the supply chain and in surveillance (i.e., diagnostic testing capacity); public-private and public-public partnerships, including the community at large; and the utilization of existing structures and systems. Some of these key drivers have been recommended as lessons to be leveraged as preparedness to future pandemics in low and middle income countries (3, 28, 29).

Regarding leadership, participants unanimously reported that this was observed in all the components of the response, particularly in establishing partnerships to help overcome the challenges faced by the heath system and in demand creation efforts, notably, the president of the republic's commitment in securing vaccines by joining the COVAX mechanism; the Minister of Health, by being the first person to be publicly vaccinated in-country and the community and religious leaders in actively mobilizing their local communities to accept vaccination. The ability of decision makers and leaders to take seriously the threat posed by health crises is crucial in responding to them (2) and this was also found to be a key driver and also a lesson learnt in many other African countries (18, 19, 30). Influencers and the media have also been involved in the response to COVID-19, in demand creation efforts, in particular, and this has been observed throughout history (31). The use of vaccination certificates has also helped to create a demand for vaccinations. However, it also created a demand for fake vaccination certificates, especially among travelers, inversely affecting infection rates. This issue was also observed in other settings (32). Additionally, the issue of certificates not being verified at home or abroad was mentioned as a point for improvement, suggesting the creation of a plan for such verification in future pandemics.

Mozambique developed a plan to address COVID-19 and to conduct vaccination efforts and was amongst the first 10 countries to do so in Africa. This allowed various stakeholders, including the community, to understand the health system's needs and identify ways to support it. The created coordination mechanism (the committee), led by the minister of health, facilitated implementation and collaboration of the different partnerships. The committee was comprised of three sub-committees: one subcommittee for planning and implementation, one for logistics and cold chain, and one for advocacy, social mobilization and communication. The existence of a national plan, a committee with a diverse representation of stakeholders, was also a determinant for an effective response in other countries (33), for example Rwanda, which had one of the lowest incidence rates of COVID-19 infection in Africa (34).

Regarding partnerships, participants were unanimous about the importance and success of partnerships in overcoming health sector challenges in responding to COVID-19. For example, the private sector contributed with about 10% of vaccines and this helped the country to vaccinate many people in a short time. Amongst aspects that have contributed to a good collaboration within the framework of partnerships was the openness of health authorities; partners flexibility; weekly virtual meetings with the technical committees; joint planning, implementation and performance evaluation; and the existence of data-sharing agreement between the public and private sectors. Coordinated public-private sector efforts including the pre-existence of communication mechanisms is essential for an integrated response during crises (7, 35).

The use of evidence for decision making was consistent across the response to COVID-19 and it played a crucial role. The creation of a multidisciplinary technical-scientific commission, chaired by the health minister, which advised the President of the Republic, is an example of a data-driven response. Data on the needs and concerns of individuals and groups regarding vaccines has been utilized in vaccine demand creation; data collected by the surveillance department has also been used to produce health statistics. A local evaluation also noted that the use of data for decision-making was one of the main elements for the agility of the response in Mozambique (18). In addition, evidence on risks and vulnerabilities was used to determine vaccine allocation (36). The use of evidence for decision making was one of the main lessons learnt in responding to COVID-19 in Mozambique and in other African countries (37).

The response to COVID-19, including vaccination was based on the utilization of pre-existing structures and systems i.e., the sentinel surveillance of flu-like syndromes system, facilitated the early detection of COVID-19 cases; the vaccination distribution and delivery structure at district and community levels (including the adaptability of the EPI to meet the needs of the target groups), contributed to vaccination targets to be met. A functioning community engagement structure is critical for an effective response to disease outbreaks and historically, countries that had health systems with strong ties to communities responded quickly and effectively to outbreaks (2, 38, 39), including existing structures for routine vaccination (40). Surveillance for adverse events and efficacy of vaccines was also based on the pre-existing system, which showed that those who did not receive vaccines were the ones with the severe cases of the disease. Investments in the supply chain and surveillance systems were made by scaling up transportation, storage of ultra-cold equipment, and diagnostic testing capacity and this enabled a better response.

Although the focus was on COVID-19, which has resulted in interruptions in access to health services, health authorities managed to minimize this and mitigated the COVID-19 impact. E.g., health authorities ensured the provision of health services for other diseases, including the setting up toll-free telephone call centers, through which people could call and ask for assistance. Additionally, a prevention campaign slogan that helped to reinforce prevention and encourage access to health services was used. It is important to highlight that the switch from a slogan that could have an effect contrary to prevention to one that reinforced prevention is a lesson learnt from another pandemic, i.e., HIV/AIDS, which initially used the slogan “*AIDS kills”* and later switched to *“protect yourself”*. However, more efforts could have been done to ensure access to other essential services, such as maternal and child healthcare, which have been severely affected (41, 42). In fact, “during a health crisis, attention should not be completely diverted from other essential services, as this could result in the re-emergence of disease and impede disease elimination and eradication efforts (43). Instead integration of services is recommended and has been a lesson learned in the implementation of the COVID-19 in Mozambique and other African countries such as South Sudan and Sierra Leone, which resulted with the establishment of a platform for adult vaccination into primary health care services (29). To mitigate the socio-economic impact of COVID-19, social security authorities contributed by assisting more than a million vulnerable populations, thus achieving the target set.

Although the country's response to COVID-19 has been largely successful, according to our participants, there are aspects that could be improved, namely coordination, funding, efficiency and equity.

First, regarding coordination, participants’ recommendations centered on the development of a strategic plan to respond to outbreaks and pandemics, which could be approved at the highest political level and reviewed every five years. The current plan provided guidelines for the implementation process, definition of target groups, vaccination delivery strategies, and detailed implementation phases. As an improvement to the current plan, a clear budget and a communication plan should be added. In addition, although most decisions were based on evidence, there were concerns regarding political influence in some of the decisions made by the COVID-19 committee, which created bottlenecks for the COVID-19 response. Furthermore, concerns were raised about inefficiencies in the vaccine distribution process, attributed by some participants to coordination failures on the part of the committee, and by others to the supply of vaccines close to their expiry date by donors. Since vaccines donated by many donors were at risk of expiry considering the short shelf life on delivery, the authorities had to accelerate distribution even if this implied higher costs. The need to supply vaccines with an adequate time frame was identified as a lesson learned for international collaboration during health emergencies, therefore suggesting that donations should be predictable and reliable. Concerning accountability in the use of funds, this could have compromised the response to COVID-19 since part of these fundsʹ application was unjustified and non-transparent. Therefore, continuous oversight to ensure that there is accountability among leaders and corruption prevention, was a lesson learnt in Mozambique and other African countries (37).

Second, as far as funding is concerned, there must be an emergency fund, because its existence is crucial to simplify the bureaucracy associated with applications for funds and therefore speed up allocation decisions. Although there was support from donors and partners, funding was insufficient (16) and there have been delays in the remuneration of retired technicians and technical assistants who have been mobilized to support vaccination (18). The lack of COVID-19 vaccine funding visibility also contributed to inefficiencies including duplication of efforts. This was also experienced in other countries (29). Financial support for the community and CSOs involved in the response is also an issue to be addressed.

Third, regarding the vaccine distribution processes, there is a need for its optimization. The decentralization of vaccine distribution to health facilities is underway, to reduce costs, reduce stoppages and allow for greater fluidity and speed in distribution, and should be reinforced, along with the high-quality, high-capacity storage infrastructure.

Fourth, the following aspects regarding equity were identified:

Regarding vaccine demand generation, interventions did not accommodate the needs of people with auditory disability and those who could not communicate in Portuguese. For example, the initial provision of information did not include sign language, including the president's communication to the nation; however, this was later addressed. Participants also highlighted the need to use local languages and some foreign languages, since most of the population is fluent in these languages and there are refugees in the country. Similarly, people with physical disabilities and the elderly (who did not live in nursing homes or did not have an escort) did not have easy access to vaccination posts. Efforts should be made to implement strategies that are suitable for these populations, such as mobile and door-to-door brigades. Addressing this is important to ensure effective health communication and access to vaccination.

Regarding decision making, vulnerable and marginalized populations were not included in the debates related to the design and implementation of the response. A local studies also found that the government's decision-making mechanisms about the COVID-19 pandemic failed to include civil society stakeholders and lacked official mechanisms of consultation, including a CSO representative in the COVID-19 technical-scientific committee (17, 19). Therefore, health authorities must ensure that all segments of the population, including the elderly, people with disability, rural and socio-economic less privileged individuals are reached by health interventions during emergencies and are represented in the decision-making process. Leaving some groups behind can, apart from an increased risk of illness and death among them, make them a source of disease re-emergence. Involving all segments of the community is important to build and maintain trust, improve confidence in the government, and instill a sense of civic mindedness and self-responsibility (3, 39, 44).

Currently, data is at a level of disaggregation that prevents knowing the level of vaccine coverage for diverse groups, including vulnerable population. Therefore, disaggregation would enable verification of which groups received and which groups missed vaccination and inform the respective corrective action (45). For example, some participants mentioned that men were amongst the least vaccinated group. Administrative data supports this, pointing to a difference of 10% between the coverage of women and men (55% and 45% regarding the 68% in the general population). Women have shown higher vaccination coverage compared to men, even in the absence of guidelines for vaccinating pregnant women, which had led to reproductive health professionals not promoting vaccination among this group due to concerns about vaccine safety for fetuses. Disaggregated data and respective databases would also be useful for social security authorities to reach vulnerable populations faster during pandemics, hence facilitate impact mitigation efforts.

Regarding surveillance, rural populations were less covered in terms of testing. Therefore, there is a need for continuous scale up and decentralization of infrastructure to detect and monitor the spread of disease. This need was also identified as a lesson learnt in Nigeria, due to its limited national testing capacity (38). While the implementation of surveillance should be decentralized, e.g., ensure testing capacity at health facilities in rural areas, there are calls for the centralization of the management process, by designing and implementing systems that allow information to come from different sectors and arrive in an integrated, unified and standardized way at an institution. Such an institution would preferably be a public one, which could control the entire data management process, i.e., from the request for the test, to the issuing of the result, to the verification and follow-up of the suspected case and respective contacts. Centralization could provide accurate data by reducing double counting. The use of early warning systems to predict, detect, and understand outbreaks with historical data is also recommended. The main lesson learnt about the aspects that need to be improved, especially regarding equity and financing for the COVID-19 response, is that these must be addressed in relation to the health sector. In this way, the country can be able to strengthen its health system and move towards universal health coverage (UHC). UHC aims to achieve three main goals: equity in access to health care, the provision of quality health care and affordable health services. These advances will also be noticeable during pandemics (29, 46).

## 5 Conclusion

This study explored the perspectives and experiences of various stakeholders involved in the design and implementation of the COVID-19 response, in particular vaccination. In general the aspects that contributed to an effective response in Mozambique were the existence of a leadership with clear strategies; a functioning coordination and funding mechanism; a phased allocation of vaccines, which prioritized groups most likely to infection, serious illness and death; investment in public-private and public-public partnerships; involvement of the community in prevention efforts; and the use systems previously put in place to control disease outbreaks. To ensure a more efficient and equitable response, interventions must address amongst others, the development of a clear budget, a communication plan, creation of an emergency fund, accountability in the use of funds, decentralization of surveillance infrastructure, and access and representation issues for other vulnerable groups including the elderly not in nursing homes, people with physical and mental disabilities, the LGBTQ community and rural populations. The lessons learned from the COVID-19 response in Mozambique, which could be considered when preparing for an effective and equitable response to future pandemics, are in essence the following: there should be government leadership, a response plan, adequate resources, use of data to inform decisions, constant vigilance, a prompt response, involvement of all stakeholders and documentation of actions for continuous learning. These lessons could improve pandemic preparedness nationally and globally.

### 5.1 Limitations and strengths

We only interviewed one participant from the private sector because the others we contacted did not respond to our request to participate. Furthermore, we only interviewed the national representatives of the institutions that designed and implemented the COVID-19 response, and not the representatives in the provinces and districts. Nor did we interview the health professionals who administered the vaccines and the implementation of COVID-19 therapy and management indications. These aspects represent a limitation of the study, since the perspective of these professionals is fundamental for a more complete and accurate analysis of the response to the pandemic. Future research could better explore the perspectives of these stakeholders and conduct quantitative research using large sample sizes. The fact that we included most of the key players that designed and implemented the response to COVID-19 (including CSOs) and comprehensively assess the response, is a strength of this study and suggests that our conclusions are a good representation of what has been learned in the response to COVID-19 in Mozambique.

## Data Availability

The raw data supporting the conclusions of this article will be made available by the authors, without undue reservation.
